# Explaining individual variation in patterns of mass loss in breeding birds

**DOI:** 10.1186/1742-4682-3-20

**Published:** 2006-05-16

**Authors:** Sean A Rands, Innes C Cuthill, Alasdair I Houston

**Affiliations:** 1 Centre for Behavioural Biology, School of Biological Sciences, University of Bristol, Woodland Road, Bristol BS8 1UG, UK; 2 Department of Zoology, University of Cambridge, Downing Street, Cambridge CB2 3EJ, UK

## Abstract

**Background:**

Studies of birds have a disproportionate representation in the literature on life-history evolution, because of the (apparent) ease with which the costs and benefits can be quantified and manipulated. During reproduction, birds frequently show a highly conserved pattern of mass change and changes in mass loss during breeding have been widely considered to be a valid short-term measure of the costs of reproduction. Experimental manipulations of the breeding attempts of birds usually argue that the presence of a response shows that a cost of reproduction exists, but there is little consensus as to how the size of these costs can be measured.

**Results:**

We model this mass loss by considering how a parent can maximise its lifetime reproductive success, using a theoretical framework that is particularly suited to modelling parental care in altricial birds. If lifetime reproductive success is taken to be the sum of a parent's current and future reproductive success, we show that the exact forms of these components will influence the optimal amount of mass a parent should lose. In particular, we demonstrate that the shape of the relationship between parental investment and chick survival will lead to differing degrees of investment between parents of different initial qualities: parents with initially high levels of energy reserves could conceivably invested a lesser, similar or greater amount of resources than parents with initially low reserves, and these initially 'heavy' parents could potentially end up being lighter than the initially 'lighter' individuals.

**Conclusion:**

We argue that it is difficult to make predictions about the dependence of a parent's final mass on its initial mass, and therefore mass loss should only be used as a short-term measure of the costs of reproduction with caution. The model demonstrates that we require a better understanding of the relationship between mass loss and both current and future reproductive success of the parent, before predictions about mass loss can be made and tested. We discuss steps that could be taken to increase the accuracy of our predictions.

## Background

Most species of bird show stereotypical patterns of mass loss during their breeding period, and studies looking at these changes in body mass have been appearing in the literature since the 1930s [1,2]. There are species-specific trends of loss during the reproductive phase, which can be related to phylogeny and life-history strategies [3]. Some but not all of the loss is due to the regression of the gonads after gametogenesis [4-6]. A reduction in mass as a result of a reduction in energy reserves could have an effect on the individual's chances of surviving to reproduce in the future (and the effort that can be put into reproduction), and so has been argued to be a physical manifestation of the costs of reproduction (e.g. [7-9]). It has also been suggested that mass loss may instead be a means of reducing the costs of flight [6,10] (leading to an increase in the amount of food available to the offspring), and hence does not necessarily reflect a cost of reproduction.

Experimental studies attempt to alter some aspect of the parent's workload or reaction to its environment – by, for example, manipulating clutch size (e.g. [11]), length of the breeding season (e.g. [12]), age of the chicks in the nest (e.g. [13]), clipping parental flight feathers (e.g. [14]), or giving the parents or chicks supplemental food (e.g. [8,15]). However, little consensus seems to exist in these studies as to the functional reasons for mass loss, and how the patterns of loss resulting from the manipulations are determined [16]. The effects of the manipulation on the mass changes in the parents are typically assessed by measuring the mass of the parents at set points during the period (such as just before the manipulation, and at the end of the chick rearing period), relative to a control.

A manipulation will affect the immediate effort a parent makes. It will also affect the offspring, either directly or indirectly. The resulting changes in the investment of a parent will therefore reflect a trade-off between its current and future reproductive success [17]. In this paper we describe a simple model that suggests that if the parent is optimising its total reproductive success, then quantifiable differences (or their absence) in mass loss and final mass may occur for reasons that have not yet been explored experimentally.

## Model formulation and results

### The basic model of optimal mass loss

The following model describes the pattern of mass loss shown over a set period of the reproductive cycle. We consider the period to start at the point when the parent alters its routine in order to devote a significant portion of its time and energy to the current reproductive attempt. (The moment at which the routine changes could be defined in many species as the point at which incubation starts, whilst in birds that migrate to a distant breeding site (such as geese and ducks), it could be the point at which pre-laying feeding ends). The end of the period of interest is taken to be the point in time at which intensive offspring care ends (such as fledging, or the cessation of provisioning). The model assumes that the parent starts the period of interest at mass *x*_0_, and has achieved a mass *x *by the end of the period.

The lifetime reproductive success of the parent is considered to be the sum of two components, the current and the future reproductive success [17]. Here, the current reproductive success is taken to be dependent upon the survival of the current brood of chicks through to reproduction, and the future reproductive success of the parent is dependent upon its ability to reproduce in subsequent breeding periods (note that a parent could engage in several consecutive breeding periods within a season). We now describe how these components of fitness are dependent upon initial and final reserve levels (note that when we refer to 'fitness' below, we are referring to the lifetime reproductive success of the parent: therefore, there are current and future components to its fitness).

We assume that the greater the amount of mass lost by the parent at a given decision step, then the greater the benefit to the chicks: their reserves will increase, and therefore so will factors such as the probability of surviving a period of bad foraging, or the amount of energy available for growth. (It may seem naïve to assume that an extremely large amount of mass lost by the parents will be extremely beneficial to the chicks, but note that we are considering a simplified form of the current fitness component here – at the same time, losing an extreme amount of mass is going to be highly detrimental to the parent's own survival, and is manifest through its future fitness component.) Thus, the resulting gain from the current breeding attempt is an increasing function of the mass loss *L *= *x*_0 _- *x*, and is denoted by *b*(*L*). Note that we ignore any effects on the chicks of the final parental mass (such as mass-dependent flight costs): see the appendix for a more general case that could include these effects.

**Figure 1:**
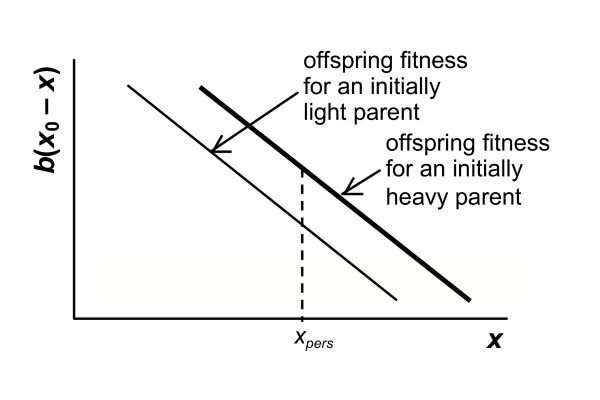
**Example of how the current fitness component, *b*(*x*_0 _- *x*), relates to final parental mass, *x***. Here, *b*(*x*_0 _- *x*) is a simple linear function of *x*. Shown are the fitness functions for parents that start off with high (heavy) and low (light) values of *x*_0 _– the resulting offspring fitness at any final mass (such as that at which future reproductive success fitness is maximised, *x*_*pers*_) is greater for the parent that starts off with higher reserves, as it has allocated a larger amount of reserves to the offspring to reach this mass.

Assuming that the fitness component from the chicks is not influenced by the mass of the parent, we could model the changes as shown in Fig. 1. With an increase in mass lost, the fitness return from the chicks increases. As the figure shows, if a parent starts off with large reserves ('heavy'), then as it approaches a set weight (such as the mass at which the parent's future reproductive success is optimised, which is defined below as *x*_*pers*_), then it will have lost more mass than an individual that started off 'light' (with low reserves), and therefore its current fitness will have reached a greater value.

We take the future reproductive success of the parent to be the success of the parent in future breeding seasons (we assume that the parent survives through the current reproductive period). This is considered to be solely dependent upon the final mass of the parent, and is denoted *p*(*x*). Consider the effects of reserve size on the future reproductive success of the parent, without considering the effects of these reserves on the chicks. The optimal parental mass, *x*_*pers*_, represents a trade-off between starvation at low energy reserve levels and greater predation risk and metabolic costs at high reserve levels [18-21]. As a consequence, it can be expected that the fitness function for parental mass should be a curve with an optimal mass occurring somewhere between maximum and minimum mass. Here, we consider the relationship to have a single maximum value. It should be noted however that for most of the arguments presented here, the *p*(*x*) function need not have a intermediate value at which it is maximised – it can be modelled simply as an increasing and decelerating function of final mass (i.e. *p' *> 0, *p" *< 0).

The parent's total fitness is considered to be the sum of current and future reproductive success. It follows that the optimal final mass x* should maximise *p*(*x*) + *b*(*x*_0 _- *x*). Differentiating with respect to final mass, it follows that x* satisfies

*p' *- *b' *= 0.     (1)

**Figure 2:**
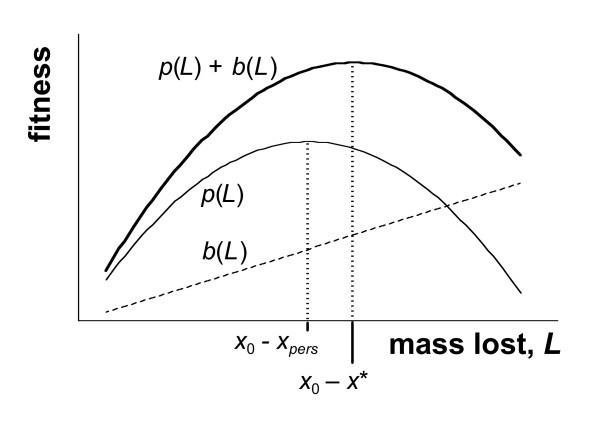
**Graph showing the optimal amounts of mass tolose**. This sketch (not to scale) demonstrates that the degree of mass lost to reach the optimal mass x* is greater than the loss necessary to reach the optimal parental mass *x*_*pers*_. Note that the shapes of the current fitness component, *b*(*L*), and the future fitness component, *p*(*L*), are arbitrarily drawn as a linear and a quadratic function here.

Because *b*(*x*_0 _- *x*) is defined as an increasing function of *L *= *x*_0 _- *x*, then *b'*(*x*_0_-x*) is positive. From (1), this means that *p'*(x*) is positive, and hence x* <* x*_*pers*_. Therefore, the final mass at which a breeding parent's lifetime reproductive success is maximised is less than the mass at which the future reproductive component is maximised (this is sketched in Fig. 2).

Implicit differentiation of Equation (1) with respect to final mass gives , which implies



where the derivatives are evaluated at x*. From Equation (1) and the second order condition for x* to be a maximum, *p" *+ *b" *< 0. This means that if *b" *is positive, then dx*/*dx*_0 _< 0; and if *b" *is negative, then dx*/*dx*_0 _> 0.

A parent's optimal loss is L* = *x*_0 _- x*. The differential of optimal loss with respect to initial mass is therefore



which, when evaluated at x*, gives



Because *p" *< 0, this tells us that a parent's optimal mass loss is an increasing function of initial reserves: parents that start with larger reserves should lose more mass overall than parents that start with lower reserves.

### The effect of the current fitness component

**Figure 3:**
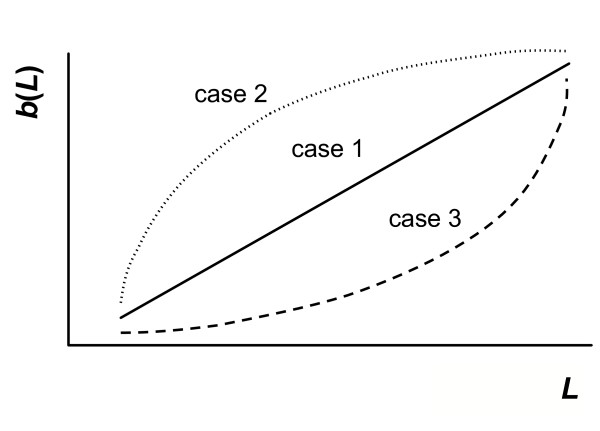
**Some possible shapes of the current fitnesscomponent, *b*(*L*)**. The figure is drawn with respect to mass lost during the period, *L*, and sketches the three cases defined in the text (*case 1*: constant slope; *case 2*: decreasing slope; *case 3*: increasing slope).

The exact shape of *b*(*x*_0 _- *x*), the current fitness component, has a great effect upon the predictions we can make about optimal mass loss in the parent. Calculating the exact benefit of mass loss (in terms of fitness) to the parent is complicated by the fact that its effects upon chick survivorship and future reproductive success are not direct. Mass loss provides extra energy to the chick [22], but the resulting value of this energy to the chick (and hence its fitness) needs to be considered as well: for example, a small amount of food may have a greater impact on the survival of a young chick in comparison to an older chick. Here, we consider some simple shapes for the current fitness component (sketched in Fig. 3), where fitness is assumed to be a monotonic increasing function of chick reserves:

*i*. constant slope (*i.e*. *b" *= 0). Gain in current fitness per unit of mass lost is constant. From Equation (2), it follows that optimal final parental mass should be constant regardless of initial mass, and so all parents should ideally be the same mass at the end of the period.

A non-constant slope could occur because the fitness of an offspring may not be proportional to its intake (*cf*. [23,24]) or because offspring intake is not proportional to mass lost by a parent.

*ii*. decreasing slope (decelerating, *i.e*. *b" *< 0). Here, although current fitness increases with increasing mass loss by the parent, the increase in fitness per unit of mass lost decreases as more mass is lost. This might be a consequence of a decrease in the ability of chicks to utilise energy as intake increases. From Equation (2), this means that the optimal final mass of initially light parents is lower than that of heavier parents, despite the heavier parents losing more mass, as shown by Equation (3).

*iii*. increasing slope (accelerating, *i.e*. *b" *> 0). Here, the increase in fitness per unit of mass lost increases as more mass is lost. This could be the case with chicks that have few stored energy reserves to rely upon, because the probability of avoiding starvation might be an accelerating function of energy intake when reserves are low (*e.g*. [23,24]). Considering initially heavy and light parents, Equation (2) indicates that in this case initially heavy parents should finish lighter than parents that start the breeding season with small reserves.

**Figure 4:**
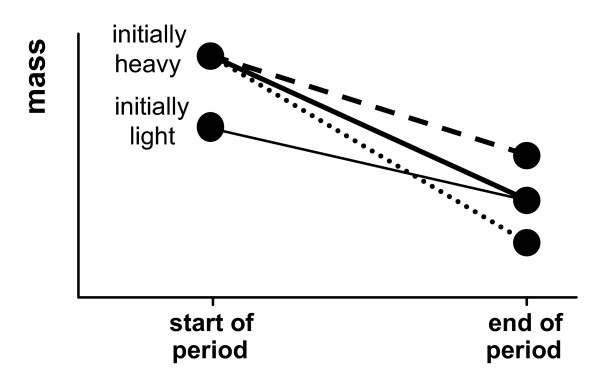
**Potential patterns of mass loss in parents withdiffering initial reserves**. Sketch (not to scale) shows the initial and final masses of an initially heavy parent in comparison to an initially light parent (note that the lines are given simply to connect initial and final masses, rather than show the shape of the mass changes during this interval; note also that the given mass loss by the initially light parent is for illustrative purposes only, and offers a benchmark mass change to compare heavy parent loss against). The line types for the initially heavy parent show the form of *b*(*L*) considered: *case 1 *– constant slope (solid line); *case 2 *– decreasing slope (dashed line); and *case 3 *– increasing slope (dotted line).

As could be expected, the parents with the largest reserves should lose the greatest amount of mass. However, as demonstrated here, the final mass of the parent depends not only on its own initial reserve level, but also on the shape of the offspring fitness function (Fig. 3). If this function has a decreasing slope (case *ii*), then patterns are as would perhaps be expected intuitively: parents that start heavy will remain heavier than initially lighter parents. Counter-intuitively, the reverse of this pattern is seen if the offspring fitness function has an increasing slope (case *iii*), initially heavier parents end up lighter than initially light parents (Fig. 4).

This is simply considering what happens when the chick reproductive success component is directly related to mass lost. We also need to consider the effects of this loss upon the parent: similar amounts of mass loss by heavy and light parents may result in them having very different flight costs, which could lead to further differences in the current reproductive success. Therefore, changes in flight costs would also have to be considered (see the appendix for a more general case where current reproductive success is related to both mass loss and final mass). Similarly, factors such as the distance travelled by the parent [22] may be important in determining the form of the parent's current reproductive success.

## Discussion

In this paper, we have described a simple model that considers mass changes in breeding birds from an adaptive perspective. The model shows that it is vital to have a clear understanding of the effects that a manipulation or environmental change has upon both the current and future reproductive success of a parent, before we can state whether an observed change in the mass of the parent reflects an optimal response to the manipulation. We have extended the established concept that mass loss may be adaptive [6,10]. We show that the shapes of functions relating the mass lost by a parent to the survival of both itself and its chicks can have subtle and counter-intuitive implications upon the patterns of loss we would see in individuals (and differences between the shapes of these functions between individuals could depend upon factors such as the age of the chicks, or individual differences between parents). Particularly, we show that it is difficult to predict the degree of mass lost by a parent with a given initial amount of energy, without giving careful consideration to both components of its fitness. In experimental studies, cases have been seen where initially heavier parents lose more mass than light parents (e.g. [25,26]). The model described here suggests that these patterns can have biological significance if the shape of the offspring fitness function is taken into consideration: for example, by considering an accelerating curve to the future fitness component as we describe here in case *iii*. It should be noted here that although we are suggesting that these patterns of mass loss can by justified biologically, care should be taken to ensure that an observed difference between individuals is not just a statistical artefact arising from estimating mass changes through regression (examples are discussed by [27,28], but see [29]). Statistical artefacts of this form are well known in other disciplines, and it is possible to avoid them with correctly designed and controlled experiments [30].

The shape of the current fitness component function may change with chick age. The main energetic expenditure of a young chick is invested in growth, and it may be the case that growth is accelerated when chick reserves are higher, which could suggest an increasing and accelerating offspring function. It may be less advantageous for older chicks to have high energy reserves (such as in species showing weight recession – see [31]), because chicks approaching fledging will have to face similar body mass trade-offs to an adult. An increasing and decelerating function may therefore be more appropriate in the case of older chicks. Therefore, it may be that the shape of the offspring fitness function changes with the age and requirements of the chicks, and so the predictions we can make are crucially dependent upon the shapes of the fitness curves (Winkler [32] also argues that the shapes of parental investment curves are important, and for a similar concept used in the discussion of food sharing and natal dispersal, see [33]). Similarly, in many passerine and non-passerine species where clutch sizes are greater than one, competition between siblings [34] may alter the forms of the fitness curves.

The exact shapes of the current fitness function (sketched in Fig. 3) are open to debate, since there is a lack of experimental evidence to suggest that one form should be favoured over the others in specific systems – in particular, the linear function (case *i*) states that all parents should end up at the same value, which is arguably unrealistic biologically, but which gives a useful null hypothesis to frame the results of our non-linear functions against. So how do we estimate the shape of this function? We could try to make inferences from related experimental work concerning the growth of chicks. We know for example that larger or heavier chicks tend to have a higher chance of surviving to reproduce [35]. Similarly, we know that if chicks are exposed to different food intakes during their development, this can affect their growth rate [36], which may in turn have effects upon their fitness [35,37]. Furthermore, we have a good understanding of the physiological constraints involved in avian development, and how this may affect the optimal growth rates of different species [38-40], which in turn gives us information about the maximum amount of investment a parent can give to a chick [41]. However, this information does not explicitly give us an obvious relationship between the amount of mass a parent loses, and the resulting increase in the survival of its chicks. This is something that still needs to be experimentally determined, preferably using techniques that will affect the survival of the chicks without potentially affecting the response of the parent (and therefore the shape of its future fitness curve, which will lead to knock-on effects regarding the optimal degree of mass to lose). This means that popular techniques such as clutch size manipulation [42-45], which definitely affect the work-load of the parent (and hence its future fitness), are not suitable for determining the shape of the current fitness component unless one can find a means of deconfounding the changes the manipulation imposes upon both the future and current fitness values.

Our model involves various simplifications, and we do not explain why parents differ in their initial reserves. This might result from some underlying difference in individual quality, or could happen through chance alone, such as a period of bad luck in finding food (or, in the case of some empirical studies, may be due to initial non-random allocation of individuals to differing experimental treatments). The timing of breeding within the season may also be important, as both chick and parental survival through to a subsequent breeding season will be affected by the date at which the chicks fledge. The timing of breeding is therefore likely to have an effect upon the optimal parental mass *x*_*pers*_, and individuals breeding earlier in the season should have differing fitness functions to those breeding later. Furthermore, the model described here considers an individual that does not die during the current reproductive attempt, and that only makes a single decision about the amount of mass it should choose to lose. Relaxing this or any of the other assumptions described would complicate the models further, and essentially confuse any predictions we could make about loss.

The model discussed is suitable for explaining loss in central-place foraging passerines and other altricial birds, where parents show most of their loss during the period when they are foraging for the chicks. A different patterns of loss is seen in birds with precocial development [3]. In most of these, one of the parents (usually the female) shows the greatest loss of mass during incubation, when the nest has to be tended constantly to keep the eggs warm and guard them from predators. On hatching, the chicks have some degree of independence, and the parents are able to forage to supplement their own reserves. This great difference in allocation strategies has led to a divide in the theoretical treatment of reserve allocation, where workers on mostly-altricial taxa have pursued very different lines to those working in mostly-precocial taxa, such as the wildfowl, where a much greater emphasis is placed upon 'capital' and 'income' strategies of investment [46]. We argue that the model developed here is flexible enough to be applied to precocial species, because it is still necessary to consider the consequences of the parent's behaviour upon the fitness of both it and the offspring. For example, for species that don't feed (or forage very little) during incubation, such as common eiders *Somateria mollissima *[47], it could be argued that the longer a parent stays on the nest, the faster the development of the embryos is going to be, and so offspring fitness will be an increasing function of mass lost (see [48] for discussion and references). Furthermore, where the parent is not able to feed (through both the demands of incubation, and the lack of food within or near the incubation environment), it is likely that energy reserve usage has a much greater effect upon its survival and future reproductive success. The parent's own future fitness is also affected by the amount it loses, and could perhaps be approximated to an increasing function of final mass (so a parent will benefit from losing as little mass as possible when it is at the breeding site), rather than a curve with an intermediate maximum value, as discussed in the model. If we were to consider a linear decrease in future fitness using the model we describe here, we are stating that a parent behaving to maximise its future fitness should lose no mass, such that *x*_*pers *_= *x*_0 _(we assume that the parent is unable to gain mass when incubating, as it does not leave the nest to feed). From Equation (1), we have shown that x* <* x*_*pers*_, and so the optimal final mass of an incubating precocial species such as the common eider will be less than its initial mass, where again the shape of the current fitness function will be important in determining the amounts of mass lost by individuals of differing qualities. Field studies have shown that the final mass of incubating parents in precocial species can be highly dependent upon initial mass, with initially heavy parents ending as the heavier individuals (*e.g*. [45,47]). This suggests that species such as common eiders may have a type *ii *current fitness curve. However, it could instead be argued that in precocial species nesting in harsh environments, the window of time in which egg-laying can occur and the massive reduction in activity during incubation mean that the time spent incubating and the rate of energy expenditure during this period are practically independent of mass – with this independence, we would therefore expect all birds to lose approximately the same amount of mass, meaning that initially heavy birds will still remain the heaviest. In precocial species, it is also likely that the shape of the parent's curve is more complex than discussed in the model, and could change with season, availability of food in the environment, predation risk, and so forth. An understanding of these functions could give an insight into the decisions made by the precocial parent – such as when and how frequent recesses away from the nest should be, and when the parent should abandon its offspring and ensure its own survival.

## Conclusion

Therefore, the model we present here is a first step towards producing a life-history model that would include temporal changes in the two fitness components in relation to chick age and time of breeding. In order to test the predictions of this current model, we would require fieldworkers to quantify both current and future reproductive success in order to assess how the shape of these functions determines the mass changes within their species. This is arguably difficult, complex, and likely to be extremely sensitive to environmental variation. In the meantime, we could instead look towards studies that accurately quantify the changes in mass within individuals at set stages in the breeding cycle [2], and address how individual variation in the survival and mass of both parents and chicks affects fitness. Given the added complexities of considering temporal changes throughout the breeding cycle and season, this is something that would then ideally be modelled using a state-dependent approach, such as through dynamic programming [24,49]. Here we could then consider parents as making multiple consecutive decisions about how much of their reserves they should allocate to both themselves and their offspring.

## Appendix: effects of final mass on optimal mass and fitness components

In the case considered in the text, *b *is a function of *L*, where *L *= *x*_0 _- *x*. In this appendix, we consider a more general case where the current fitness gain is a function *B *of *x *and *x*_0_. In this case, optimal final mass x* should maximise *p*(*x*) + *B*(*x*, *x*_0_), which occurs when



Implicit differentiation of this equation with respect to *x*_0 _when *x *= x* gives



which, when rearranged, gives



Predictions from this case are similar to those given in the main text: because the term *∂*^2^*B*/*∂x∂x*_0 _can justifiably be positive, zero or negative, we cannot predict the pattern of loss without first how *B *depends on *x *and *x*_0_.

## Competing interests

The author(s) declare that they have no competing interests.

## Authors' contributions

This model was formulated by SAR and refined by SAR, ICC and AIH. SAR wrote and edited the manuscript, which was read and approved by all authors.
